# Whole body irradiation with intensity-modulated helical tomotherapy prior to haematopoietic stem cell transplantation: analysis of organs at risk by dose and its effect on blood kinetics

**DOI:** 10.1007/s00432-023-04657-7

**Published:** 2023-03-01

**Authors:** Mümtaz Köksal, Jonathan Baumert, Danny Jazmati, Felix Schoroth, Stephan Garbe, David Koch, Davide Scafa, Gustavo R. Sarria, Christina Leitzen, Gregor Massoth, Achilles Delis, Annkristin Heine, Tobias Holderried, Peter Brossart, Thomas Müdder, Leonard C. Schmeel

**Affiliations:** 1 Radiation Oncology, University Hospital Bonn, Bonn, Germany; 2 Radiation Oncology, University Hospital Düsseldorf, Düsseldorf, Germany; 3 Anaesthesiology, Perioperative and Pain Medicine, University Hospital Bonn, Bonn, Germany; 4 Internal Medicine-Oncology, Haematology and Rheumatology, University Hospital Bonn, Bonn, Germany

**Keywords:** Blood kinetics, Total body irradiation, Helical tomotherapy, Dose sparing, Bone marrow transplantation, Acute lymphocytic leukaemia, Acute myeloid leukaemia, Neutral killer cell lymphoma

## Abstract

**Background:**

Intensity-modulated helical tomotherapy (HT) is a promising technique in preparation for bone marrow transplantation. Nevertheless, radiation-sensitive organs can be substantially compromised due to suboptimal delivery techniques of total body irradiation (TBI). To reduce the potential burden of radiation toxicity to organs at risk (OAR), high-quality coverage and homogeneity are essential. We investigated dosimetric data from kidney, lung and thorax, liver, and spleen in relation to peripheral blood kinetics. To further advance intensity-modulated total body irradiation (TBI), the potential for dose reduction to lung and kidney was considered in the analysis.

**Patients and methods:**

46 patients undergoing TBI were included in this analysis, partially divided into dose groups (2, 4, 8, and 12 Gy). HT was performed using a rotating gantry to ensuring optimal reduction of radiation to the lungs and kidneys and to provide optimal coverage of other OAR. Common dosimetric parameters, such as D05, D95, and D50, were calculated and analysed. Leukocytes, neutrophils, platelets, creatinine, GFR, haemoglobin, overall survival, and graft-versus-host disease were related to the dosimetric evaluation using statistical tests.

**Results:**

The mean D95 of the lung is 48.23%, less than half the prescribed and unreduced dose. The D95 of the chest is almost twice as high at 84.95%. Overall liver coverage values ranged from 96.79% for D95 to 107% for D05. The average dose sparing of all patients analysed resulted in an average D95 of 68.64% in the right kidney and 69.31% in the left kidney. Average D95 in the spleen was 94.28% and D05 was 107.05%. Homogeneity indexes ranged from 1.12 for liver to 2.28 for lung. The additional significance analyses conducted on these blood kinetics showed a significant difference between the 2 Gray group and the other three groups for leukocyte counts. Further statistical comparisons of the dose groups showed no significant differences. However, there were significant changes in the dose of OAR prescribed with dose sparing (e.g., lung vs. rib and kidney).

**Conclusion:**

Using intensity-modulated helical tomotherapy to deliver TBI is a feasible method in preparation for haematopoietic stem cell transplantation. Significant dose sparing in radiosensitive organs such as the lungs and kidneys is achievable with good overall quality of coverage. Peripheral blood kinetics support the positive impact of HT and its advantages strongly encourage its implementation within clinical routine.

## Introduction

Total body irradiation (TBI) is an integral component of allogeneic bone marrow transplant (BMT) conditioning regimens, alongside chemotherapy, and is used both as a myeloablative treatment and as a tool to eradicate residual malignant cells after chemotherapy (Sabloff et al. 2021; Wong et al. 2018). The challenge is to find the optimal dose to eradicate all malignant cells to prepare the bone marrow for haematopoietic stem cell transplantation (HSCT), while keeping the toxic and adverse effects of radiation to an acceptable minimum. TBI has been shown to effectively treat diseases, such as acute lymphoblastic leukaemia (ALL), acute myeloblastic leukaemia (AML), and neutral killer cell lymphoma (Ringdén et al. 2009; Marnitz et al. 2014; Maeng et al. 2017). While there are several alternatives for delivering radiation, TBI using intensity-modulated helical tomotherapy (HT) improves the stability and homogeneity between a planned and delivered dose and also simplifies the whole process of TBI delivery (Hui et al. 2005; Peñagarícano et al. 2011). Several clinical trials have now demonstrated the feasibility and potential of intensity-modulated helical tomotherapy in preparation for HSCT (Schultheiss et al. 2007; Shueng et al. 2009; Wong et al. 2009).

At our institution, HT is used to irradiate patients as part of haematopoietic stem cell transplantation. Although HT is beneficial for these patients, BMT is still associated with serious risks and is often associated with systemic effects. A variety of organs can be damaged by radiation. These organs at risk (OAR) need to be considered when planning HSCT and especially when planning TBI. Cox et al. summarised the effect that radiation can have on a patient's OAR when receiving any form of radiation, including TBI (Cox et al. 1995).

In this study, we aim to investigate the dosimetric quality of OAR, such as lung, chest, liver, kidney, and spleen, and highlight potential differences between follow-up parameters comparing different prescribed doses. Lung sparing while maintaining good-quality chest coverage has been demonstrated in the previous studies (Wilhelm-Buchstab et al. 2020; Gruen et al. 2013). As part of this investigation, we intend to investigate the possible effects of reducing the lung dose while maintaining good overall coverage on radiation-induced lung injury. The kidneys are highly exposed during intensive HSCT, especially considering the wide variety of chemotherapeutic agents that can cause renal dysfunction in addition to the effects of radiation (Sawinski 2014; Kal and Kempen-Harteveld 2006). The spleen plays an important role in many aspects of the human haematopoietic and lymphatic systems. Particularly, in diseases, such as ALL, AML, or NK cell lymphoma, the spleen needs to be considered and focused on (Alexandru et al. 2021; Chin et al. 2018; The spleen as an organ at risk in paediatric radiotherapy: A SIOP-Europe Radiation Oncology Working Group report.Available from:https://pubmed.ncbi.nlm.nih.gov/33271483/ xxxx). To assess the impact of TBI in the preparation of HSCT, the analysed peripheral blood kinetics, such as leukocytes, neutrophils, haemoglobin, and platelets, can be associated and analysed. In addition, overall survival (OS) and graft-versus-host disease (GvHD) were included to further evaluate the impact of TBI use in HSCT. Whenever radiation is used, dose plays an essential role. Therefore, we included dose-dependent groups to evaluate the effects and their magnitude in relation to a prescribed dose. We are trying to combine the analysis of OAR quality of coverage with HT with the possible effects and toxicities that this irradiation may have and put them in the perspective of a prescribed dose. This study aims to provide an overview of the challenges and opportunities of TBI using intensity-modulated helical tomotherapy, using dose and dose sparing, quality of coverage and homogeneity, as well as peripheral blood kinetics and related data.

## Methods

This study was performed for routine quality assurance and complies with the German Radiation Protection Act, which is the standard of care at our institution. Therefore, ethical approval was not required.

### Patients

Data from all patients who received TBI in preparation for HSCT at our institution between 2012 and 2020 and who received irradiation using Helical Tomotherapy with the Hi-ART II were included. We retrospectively extracted and analysed following data: prescribed dose, dose quality of liver, spleen, kidney, lung and rib coverage, fractionation of TBI, haemoglobin, neutrophils, leukocytes, platelets, creatinine clearance, glomerular filtration rate (GFR), graft-versus-host disease (GvHD), and overall survival (OS).

### Treatment planning

Hi-ART II TomoTherapy® is limited to a maximum table movement of 135 cm. Therefore, two planning CTs were acquired for all patients with a height greater than 135 cm, which in our case included all patients in this study. These CT scans were performed using a head-fixation mask and a vacuum mattress ensuring no change in patient positioning between two planning CTs and treatment. A cranial CT is performed in cranio-caudal orientation, and a caudal CT is performed in a caudo-cranial orientation, each with a slice thickness of five mm. Scans are then after aligned, as are the treatment plans, using a radio-opaque marker placed on the patient's thigh. Exact placement depends on the patient's height, with the main restriction that none of the two scans should be more than 135 cm apart. Further technical considerations are taken to ensure optimal overlap in the junction area, with minimal over- or under-dosing and maximum robustness to changes in patient position, to correctly match treatment plans (Köksal et al. 2022). Above-mentioned CT scans and associated treatments were planned, delineated, aligned, and fused using an Eclipse treatment planning system (Varian Medical System, Palo Alto, CA). A field width of 2.5 cm, a pitch of 0.390, and a planned modulation factor of 2.7 were used as planning parameters. Some of the organs at risk analysed were delineated using a standardised template, e.g., lungs and kidneys, spleen, liver, and thorax, were delineated individually. All delineations were then customised for each patient. Average beam-on time for both treatment plans was 39.23 min. However, a 90-min time window is allowed for each patient. The extra time was used for preparation before and between treatment plan delivery and further discussion if necessary. Patients in this study received either a total dose of 2, 4, 8, or 12 Gy divided into 2 Gy single fraction. Because fractionation and total dose can affect toxicities and quality of coverage, patients were allocated into dose-related groups for some of the following statistical calculations (Shinde et al. 2019; Carruthers and Wallington 2004; Bolaños-Meade et al. 2019). Dose delivered to lungs was reduced in our treatment plans to reduce the risk of radiation-induced lung injury and diseases such as pneumonitis (Wilhelm-Buchstab et al. 2020; Carruthers and Wallington 2004). Both kidneys also received a dose reduction in all cases delivering a total dose of 4, 8, or 12 Gy, with the exception of patients obtaining 2 Gy. Liver and spleen did not receive any dose reductions in our treatment plans.

To analyse the quality of coverage, commonly used parameters, such as D95, D50, and D05, were recorded and later analysed (Wong et al. 2018). In addition, the homogeneity index was calculated using the formula postulated by Kataria et al. (HI = D05/D95) (Kataria et al. 2012).

Further qualifying these findings, peripheral blood parameters were analysed for a follow-up period of 50 days after radiotherapy. All other data were collected until 2021 or until patient's death. GFR and creatinine are used to classify renal function. Neutrophils, leukocytes, and platelets were used to monitor effects on spleen function. Haemoglobin (Hb) levels were also examined to assess the effect of irradiation. All peripheral blood parameters were measured directly, except for GFR, which was calculated using the MDRD4 formula. In addition, simple one-way ANOVA was used to detect statistical differences between patients receiving different doses. All ANOVAs were calculated at *p* = 0.05 and 95% confidence interval.

All statistical calculations were performed using SPSS v28.0 (IBM, Armonk, New York, USA) and GraphPad Prism (v.9.5.0).

## Results

A total of 46 patients were included in this analysis, all of them received TBI as part of a BMT regimen at our institution between 2012 and 2020. Their mean age at the time of their first irradiation cycle was 45 ± 17.227 years, with the youngest being 13 years and the oldest 72 years. Of 46 patients included, 21 were male and 26 were female (Table 1). Most common primary diagnose indicating HCST and TBI was AML (Alexandru et al. 2021) and ALL (Köksal et al. 2022). Less common primary diagnoses are displayed in the patient characteristics table. The majority of patients received a total prescribed dose of 8 Gy (Alexandru et al. 2021). Only 8 patients received 2 Gy, representing the smallest group.

**Table 1:** Patient characteristics

Sex	Male	21
	Female	25
Age (years)	Average ± SD	45 ± 17,22
Diagnosis		53
	ALL	19
	Mastcell leukaemia	1
	MPAL	2
	Idiopathic myelofibrosis	1
	DLBCL	1
	AML	16
	CML	1
	MDS	4
	ALCL	1
Dose (Gray)	2	8
	4	9
	8	16
	12	13

**Fig. 1:**
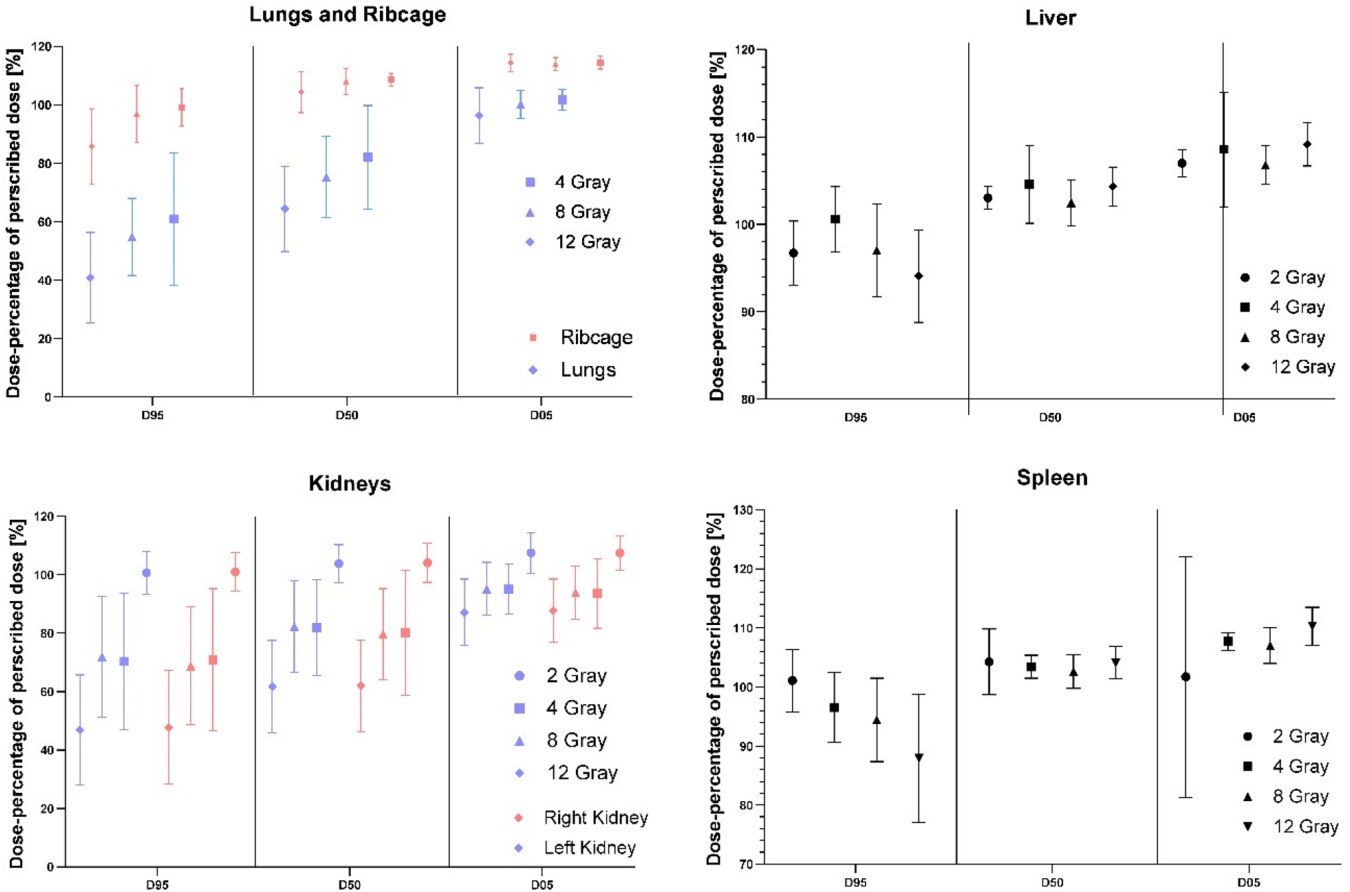
Dosimetric analyses displaying D_05_, D_50_, and D_95_ of the Lungs and Ribcage (top left), the Liver (top right), the Kidneys (bottom left), and the Spleen (bottom right). Displayed are the dose groups (2, 4, 8, and 12 Gray). Relative dose as percentage of prescribed dose is displayed on the y-axis

Dosimetric analysis of the chest and lungs is characterised by a slightly lower D95 compared to D50 and D05, respectively, achieved by the intended dose sparing during planning (Table 2; Fig. 1). Mean D95 of the lung is 48.23%, less than half of the prescribed unreduced dose. The D95 of the chest is almost twice as high at 84.95%. The averages for maximum dose received by 5% of the delineated area, D05, are closer with a difference of only about 7%. When comparing different groups in terms of dose sparing efficiency, there are significant differences in all groups when comparing chest and lung doses, except for D05.

**Table 2:** Average, minimum, maximum, and standard deviation of D_95_, D_50_, and D_05_ for all organs at risk analysed

	Average	Min	Max	SD
Lungs				
D_95_	48,23	23,00	79,76	16,43
D_50_	69,86	38,62	99,00	15,18
D_05_	98,87	69,78	107,00	6,77
Ribcage				
D_95_	84,95	57,32	100,93	11,26
D_50_	98,45	78,50	106,11	5,25
D_05_	105,76	100,93	114,00	2,34
Liver				
D_95_	96,79	86,87	109,32	5,03
D_50_	103,47	96,00	114,00	2,74
D_05_	107,81	101,00	121,00	3,27
Spleen				
D_95_	94,29	71,72	113,00	8,71
D_50_	103,38	94,00	117,00	3,31
D_05_	107,05	53,27	122,00	9,14
Right kidney				
D_95_	68,64	23,04	114,00	25,18
D_50_	78,92	32,00	118,00	20,31
D_05_	94,27	67,38	120,00	11,27
Left kidney				
D_95_	69,32	28,16	115,00	25,26
D_50_	80,08	50,54	117,00	19,61
D_05_	94,92	68,59	122,00	11,039

All prescribed doses for each OAR are combined in this table

Liver quality of coverage parameter demonstrates a slight decrease with increasing prescribed dose (Fig. 1). There is also a decrease in the standard deviation (SD) with increasing dose. Overall values range from 96.79% for D95 to 1078% for D05 (Table 2). Homogeneity indices range from close to 1 in OAR without dose reduction, to about 1.53 and 1.54 in both kidneys, and to over 2.28 in the lungs (Table 3). ANOVAs comparing all dose groups for the quality of coverage for all OARs show no significant differences.

**Table 3:** Homogeneity index for each OAR

	Average	Min	Max	SD
Liver	1,1164	1,0061	1,2500	0,06,160
Right kidney	1,5440	1,0163	3,0556	0,5379
Left kidney	1,5377	1,0211	3,0000	0,5300
Spleen	1,1468	0,5271	1,5140	0,1569
Lungs	2,2760	1,2941	4,1739	0,7512
Ribcage	1,2672	1,0588	1,7609	0,1764

Figure 1 also shows renal dosimetric values with a decrease in values, especially in D95, increasing with prescribed dose similar to the lungs. Average dose sparing of all patients analysed results in an average D95 of 68.64% in the right kidney and 69.31% in the left kidney. While maximum dose D05 also decreases with increasing prescribed dose, values of 94.27% in the right and 94.92% in the left kidney are presented in Table 2.

Finally, dosimetric values of the spleen are displayed in Fig. 1, with stable values for all parameters and prescribed doses. However, a higher SD is shown for D05 in the 2 Gy group (Fig. 1; Table 2). Significance tests (ANOVAs) performed to determine statistically relevant differences between the parameters of splenic quality of coverage show no statistical significant effect.

**Fig. 2:**
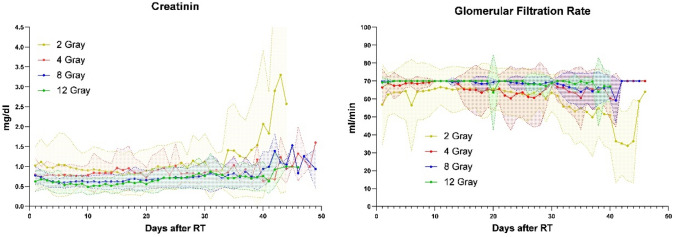
Creatinine (left) and glomerular filtration rate (right), comparison between four dose groups over the follow-up duration of 50 days. The transparent areas represent 1 SD over and below the average value

In addition to these dosimetric analyses, blood kinetics were extracted from the patient data over the follow-up period of 50 days after radiotherapy. Patients were assigned to their respective dose groups (2, 4, 8, and 12 Gy) to examine possible differences in effects when analysed by dose. In the case of the kidneys, the parameters also take into account the importance of dose reduction. Graphs of GFR and creatinine were generated to visualise renal function (Fig. 2). It is important to note that the highlighted background indicates one standard deviation (SD) above and below each mean to capture most outliers. An increase after about 40 days, a doubling of the values, can be observed in all groups and even more so in the 2 Gray group. Looking at the GFR graph, a similar observation can be made for a decrease from 10 to 30 ml/min. In addition, an increase back to 70 ml/min can be observed at the end of the follow-up period. Simple one-way ANOVAs were performed to specifically measure any significant deviation between the dose-group data. For creatinine, there were no significant differences between 4, 8, and 12 Gray, whereas the 2 Gray group differed in terms of higher values, with p values below 0.05 compared to all other groups individually. A similar aberration can be observed in the GFR ANOVA, i.e., a significant decrease in GFR values in the 2 Gray group compared to the other groups.

**Fig. 3:**
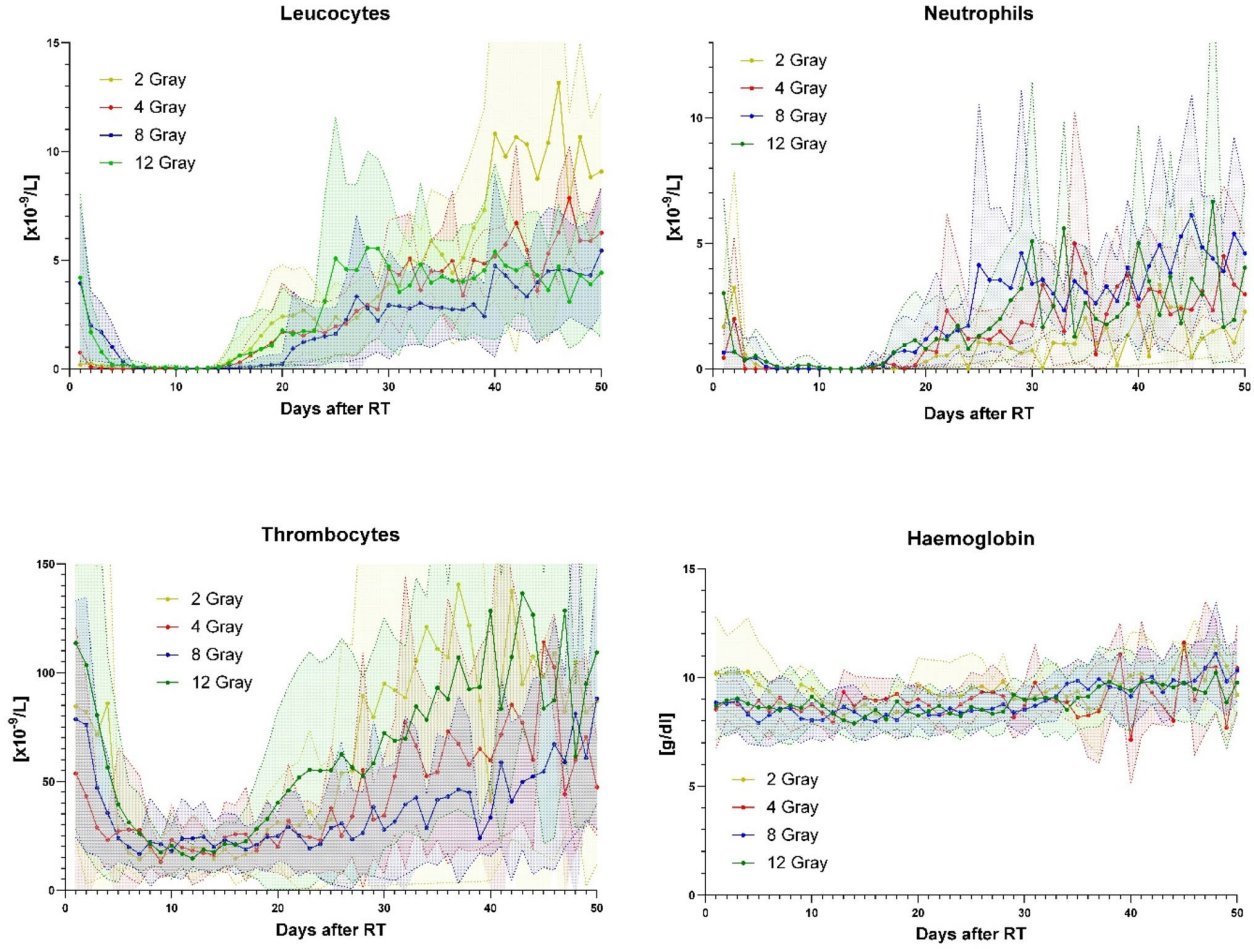
Leucocytes, neutrophiles, thrombocytes, and haemoglobin counts over 50 days follow-up after last radiation therapy. Dose groups are itemized by colour. Transparent areas represent 1 SD over and below the average value

To facilitate the dosimetric values of the spleen, the numbers of leucocytes, neutrophils, and thrombocytes are shown for 50 days after RT (Fig. 3). In all three cases, an initial decrease after RT is followed by a renewed increase after 17 days (leucocytes) and 30 days (neutrophils and thrombocytes). The additional significance analyses performed on these blood kinetics showed a significant difference between the 2 Gray group and the other three groups for leukocyte counts. Although there were significant differences between each group, these were only for individual values. Independently, the haemoglobin kinetics were also examined for significant differences among the four groups (Fig. 3), showing no significant abnormalities except for single values. No significant differences were found in the overall curve shape.

Finally, further ANOVA tests were performed to examine overall survival, graft-versus-host disease (GvHD), particularly in the liver, and cause of death. All of these showed no significant differences between the dose groups. Additional data can be found in the Supplementary Appendix.

## Discussion

TBI contributes positively to BMT regimens and is a well-established modality for treating diseases requiring haematopoietic stem cell transplantation (Cahu et al. 2016). In preparation for HSCT, irradiation is often used to suppress the patient's own immune system and eradicate any malignancy in the patient's blood and haematopoietic tissues (Stein and Forman 2008). Although these systematic treatments result in high levels of toxic effects, TBI is a viable approach for reducing these toxic effects compared to other radiation methods (Felice et al. 2016). Helical TomoTherapy® can further reduce the toxicities caused by radiotherapy. Therefore, in this study, we aimed to investigate the benefits of HT to potentially increase the advantages that this treatment option already offers and to demonstrate the opportunities and challenges associated with it.

In the OAR lung, it is of utmost importance to minimise radiation exposure as the tissue is highly sensitive to radiation (Marks et al. 2010; Oertel et al. 2021 Jun 12). However, several clinical studies, including this one, have shown that lung sparing with HT is feasible (Wilhelm-Buchstab et al. 2020; Schröder et al. 2020; Köksal et al. 2022). In our patient cohort, none had acute (< 6 months) severe radiation-induced lung injury, suggesting a beneficial effect of lung dose reduction, despite the fact that certain chemotherapeutic agents, such as fludarabine-containing regimens, are impacting on pulmonary outcome (Oertel et al. 2021). The current study highlights previous findings at our institutions, suggesting that lung dose reduction with HT is feasible and may reduce the risk of adverse pulmonary effects while not significantly compromising the quality of thoracic coverage (Köksal et al. 2022).

The data we gathered regarding the liver suggest that HT provides good coverage of the entire PTV with only a small SD and range. The HI of 1.11 was the closest to the optimal value of 1 among the HIs we calculated in this study (Cox et al. 1995). However, the liver is quite resistant to radiation and can withstand a relatively high dose of radiation before being seriously damaged (Takamatsu et al. 2018; Benson et al. 2016). However, the importance of Graft-versus-Host Disease (GvHD) in the liver after HSCT suggests that optimal irradiation of this OAR should be pursued to neglect any negative effects of radiotherapy on it (Strasser et al. 1999; Xhaard et al. 2012). Nine of the 46 patients included in this study suffered from GvHD with a focus on the liver, ranging in severity from grade I to grade IV. Although significance tests did not show a correlation between liver GvHD and the dose administered, a dose bolus to the liver could be considered. Further studies are needed to evaluate whether a dose bolus reduces GvHD with a focus on the liver, while further increasing the toxic effects of HT on the organ.

Another OAR we have analysed is the kidney. It is important to note at the outset that we did not apply dose reductions to the kidneys in patients receiving 2 Gy. However, our data suggest that HT is a feasible tool to reduce the dose delivered to the kidneys. The reduced D95 values of 68.64% and 69.32%, respectively, show a reduction of almost 30%, which corresponds to a quantitative dose reduction of up to 3.5 Gy for treatment plans with a prescribed dose of 12 Gy. The increased HI is partly due to the dose reduction and is still within the guideline recommendation (Cox et al. 1995). In terms of creatinine and GFR, the data suggest a toxic effect occurring around 40 days after radiotherapy. However, there are many variables that can influence renal function, such as the primary disease, chemotherapeutic and other drugs, or even pre-existing kidney disease (Kogon and Hingorani 2010). Nevertheless, the decline in renal function after about 40 days may be partly due to radiotherapy. However, it is more likely that the factors mentioned above are more important. Nevertheless, this suggests that it is important to minimise the risk of radiation-induced renal injury by planning a reduced dose to the kidneys, so that the already challenging task of managing the HSCT process is not further complicated (Kal and Kempen-Harteveld 2006). In our results, there was a significant worsening of renal parameters in the 2 Gray group in contrast to the rest of the patients. This relationship between dose and renal function may suggest that further studies are warranted to provide additional information.

The spleen is also an important organ to consider when planning HSCT and TBI, as it is closely linked to the body's haematopoietic and lymphatic systems. Several parameters can indicate dysfunction, such as lymphocytes and neutrophils, which can be caused by radiation. Alexandru et al. stated that an excessively high dose delivered to the spleen may cause lymphopenia during chemotherapy (The spleen as an organ at risk in paediatric radiotherapy: A SIOP-Europe Radiation Oncology Working Group report.Available from:https://pubmed.ncbi.nlm.nih.gov/33271483/ xxxx). However, we did not observe such problems after the last cycle of radiotherapy, which did not show any significant overdosage above the threshold of 14.4 Gy as mentioned in aforementioned publication. In addition, Chadha et al. showed significant differences in OS between patients who received a mean splenic dose of 9.79 Gy and 5.98 Gy, respectively, suggesting that a dose reduction to the spleen could also be used (Chadha et al. 2017).

In addition to previous conclusions, our data suggest that HT is feasible in terms of homogeneity and good quality of coverage, with an average D95 of 94.29% ± 8.71%. Different doses received by the patients undergoing radiotherapy were not significantly diverse according to the ANOVA we performed, demonstrating that even with fractionation, the quality of coverage remains at an excellent level. Normal tissue tolerance to radiation is close to the doses we deliver. It can already show low toxic effects around 10 Gy (The spleen as an organ at risk in paediatric radiotherapy: A SIOP-Europe Radiation Oncology Working Group report.Available from:https://pubmed.ncbi.nlm.nih.gov/33271483/ xxxx). This makes it all the more important to ensure good radiotherapy planning and quality.

Hematopoietic stem cell transplantation has many challenges that need to be addressed and optimised to provide optimal care. Other studies have already investigated many areas involved in this process: for example, peripheral blood kinetics help to identify challenges. The 50-day follow-up parameters of platelets, leukocytes and neutrophils show an initial drop after irradiation, which is necessary to ensure good graft engraftment. After about 17 days, leukocytes increase, and after about 29 days, platelets and neutrophils increase, and the average values rise again. They do not reach the pre-radiation levels, which could be due to the fact that the follow-up was stopped after 50 days. However, these results suggest that it is important to be aware that suboptimal treatment planning and radiotherapy may cause problems in recovery (Alexandru et al. 2021; Chin et al. 2018). Other studies have also analysed peripheral blood kinetics and found that a period of 3 days or more between the last fraction of radiation and BMT engraftment increases the chance of successful engraftment (Dejonckheere et al. 2022).

The need for two planning CT scans and treatment planning, as well as correct matching in the connection area (e.g., to avoid over- and under-dosing) and patient movement during and between fractions, is another challenge for TBI using HT. A viable approach to this challenge is the use of a static four-field box with no dose gradient (Köksal et al. 2022). This method provides high stability and robustness to patient motion while keeping the beam-on time low.

The flexibility offered by HT provides other options for delivering radiotherapy prior to HSCT, such as total marrow irradiation (TMI). Several studies have already provided data on the feasibility and viability of TMI to significantly reduce OAR and beam-on time (Köksal et al. 2023). While TBI is still improving, TMI is a viable alternative that shows a good overall outcome in engraftment GvHD and relapse-free survival, but also presents unique challenges in treatment preparation (Haraldsson et al. 2021, 2019).

## Conclusion

To ensure the best possible treatment and to optimise TBI with HT, we performed a dosimetric analysis of OAR and highlighted possible toxicity effects. Helical TomoTherapy® exploits the ability to spare organs at risk while ensuring good overall dose coverage, providing guideline (ICRU and RTOG) compliant quality of coverage and homogeneity across all different prescribed doses. In addition, dose reduction of selected OARs ensures low levels of toxic and adverse effects without loss of therapeutic dose in the total PTV. Therefore, TBI with HT should be considered a well-established and feasible treatment option for patients in need of HSCT. HT does not increase toxic effects with regard to the analysed OAR. When used correctly, it can improve engraftment and reduce GvHD, while monitoring peripheral blood kinetics helps to adjust treatment and improve overall outcome. However, these suggested toxic effects, in addition to the residual toxicities described in several other studies, invite additional studies to further reduce the residual negative effects that TBI inevitably inherits due to the nature of the radiation itself.

## Declarations

### Availability of data and materials

All data relevant to this publication have been included into the manuscript’s body.
